# Main findings of the European Health Report 2021: Ausra Zelviene

**DOI:** 10.1093/eurpub/ckac129.131

**Published:** 2022-10-25

**Authors:** A Zelviene

**Affiliations:** Data and Digital Health Unit, WHO Regional Office for Europe, Copenhagen, Denmark

## Abstract

The EHR2021 examines the WHO European Region's progress towards the health-related SDGs, the impact of the COVID-19 pandemic on population health, and approaches to build back better from 2022 onwards. It provides insight into the main challenges related to the 3 core priorities of the EPW, which are moving towards UHC, protecting people better against health emergencies, and ensuring healthy lives and well-being for all at all ages. In this presentation, we will have look at these 3 core priorities in more detail. Although the European Region is making good progress for some of the SDG targets, challenges and delays exist across the core priorities. For example, the incidence of catastrophic health spending ranges from 1% to 19% across countries, and the European Region is one of two global regions where the overall number of new HIV infections is increasing. One year into the pandemic, 29% of health services were still at least partially disrupted in the Region. The impact of the pandemic aggravates the effort Member States will have to make to reach the health-related SDGs by 2030. Furthermore, there are large and persistent differences between Member States. The COVID-19 pandemic has exacerbated existing health inequalities by impacting vulnerable groups most severely. To build back after the COVID-19 crisis, the EPW will be the leading policy framework for the coming years to take on the challenges identified. The report shows that the evidence base for supporting the policy efforts that Member States will have to make for reaching the SDGs and working on the EPW core priorities is suboptimal. While the COVID-19 pandemic has emphasized the importance of health information and sparked innovative ways of data collection and processing, there are still data gaps for key indicators and operational challenges in HISs. Overcoming these issues in health information is critical for improving the health of the people in the WHO European Region.

